# Supplementary material for: Dynamics and quantitative contribution of the aminoglycoside 6′-N-acetyltransferase type Ib to amikacin resistance
Source: mSphere. 2024 Feb 14;9(3):e00789-23. doi: 10.1128/msphere.00789-23 (PMC10964403; doi:10.1128/msphere.00789-23)
Supplement: Supplemental figures and tables — Figures S1-S4 and Tables S1-S4. [file msphere.00789-23-s0001.pdf]

Supplementary data

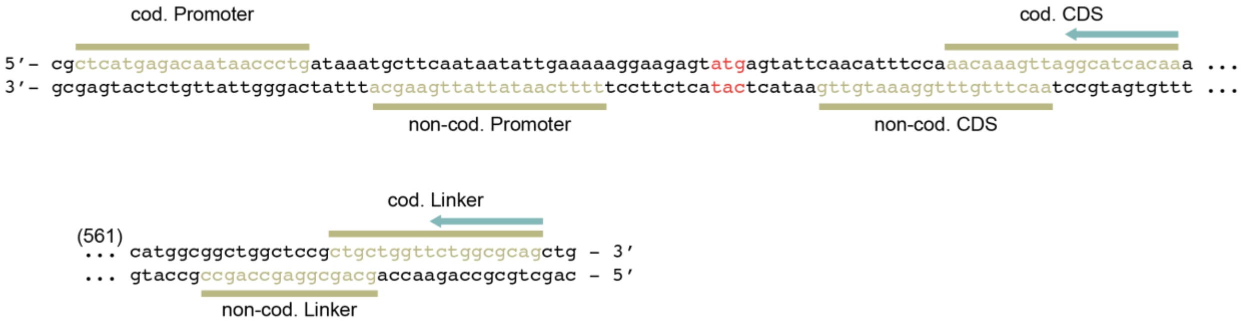

Figure S1 – dCas9 targets (Promoter, CDS and Linker on both the coding (cod.) and non-coding (non-cod.) strands) in the *aac(6')-Ib* gene on the pJHCMW1-derivative plasmid. The complementary sequence to the gRNA (green bar) is highlighted in green and the blue arrow represents the positions at which the mismatches in the sequence of the gRNA were introduced. The *aac(6')-Ib* START codon is indicated in red and the number of base pairs not included in this representation are indicated in parenthesis.

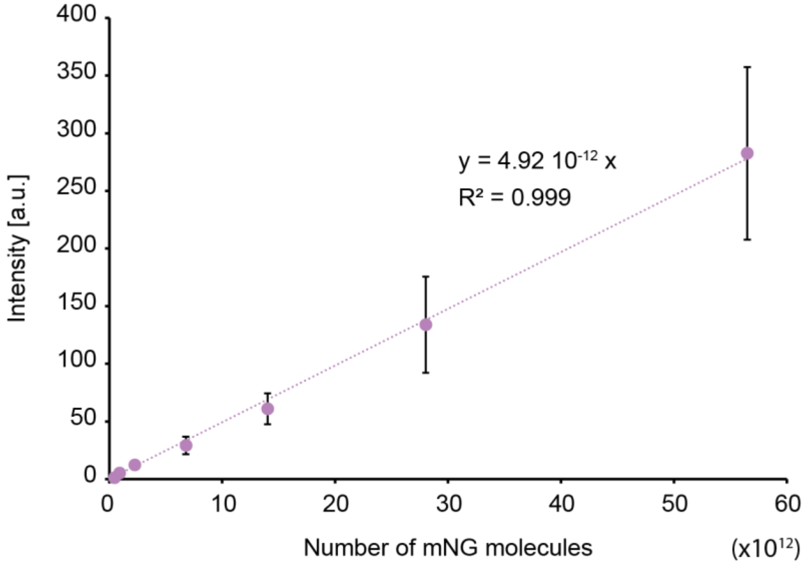

Figure S2 – Calibration curve between mNeonGreen intensity measured by spectrofluorometry (in arbitrary units, a.u.) and the number of mNeonGreen molecules. The equation of the linear trendline, as well as the correlation coefficient (R^2), are indicated on the plot.

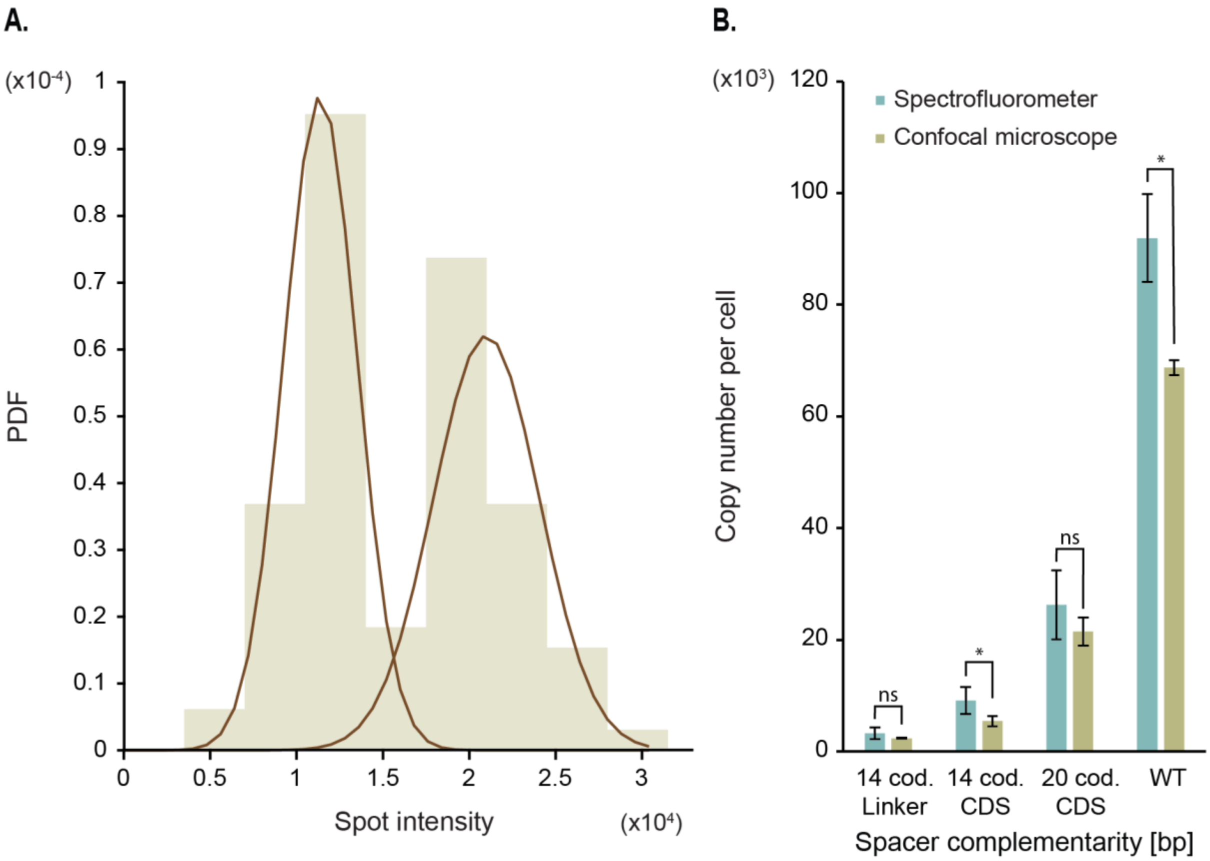

Figure S3 – Confirmation of AAC(6′)-Ib copy number by confocal microscopy. **A.** mNeonGreen intensity per molecule, estimated by imaging Nup59-mNeonGreen. The brown lines represent the Gaussian Mixture Model fitted to each population. The one on the left represents the 16-mer Nup59 (mean intensity = 11374.4), and the second peak carries 32 Nup59 (mean intensity = 20988.6). This gives an average intensity of 710.9 per mNeonGreen molecule. In total, 93 spots are represented in this plot. **B.** AAC(6′)-Ib copy number estimated with the spectrofluorometer (blue bars) and with the confocal microscope (green bars) for four strains, each carrying a different guide RNA. The length of the gRNA that is complementary to the coding (cod.) strand, as well as the targeted region of the *aac(6′)-Ib* gene, are indicated. Error bars represent the standard deviation and comparative statistical analysis was performed to compare the two methods (ns, not significant, and *, significant for p=0.05).

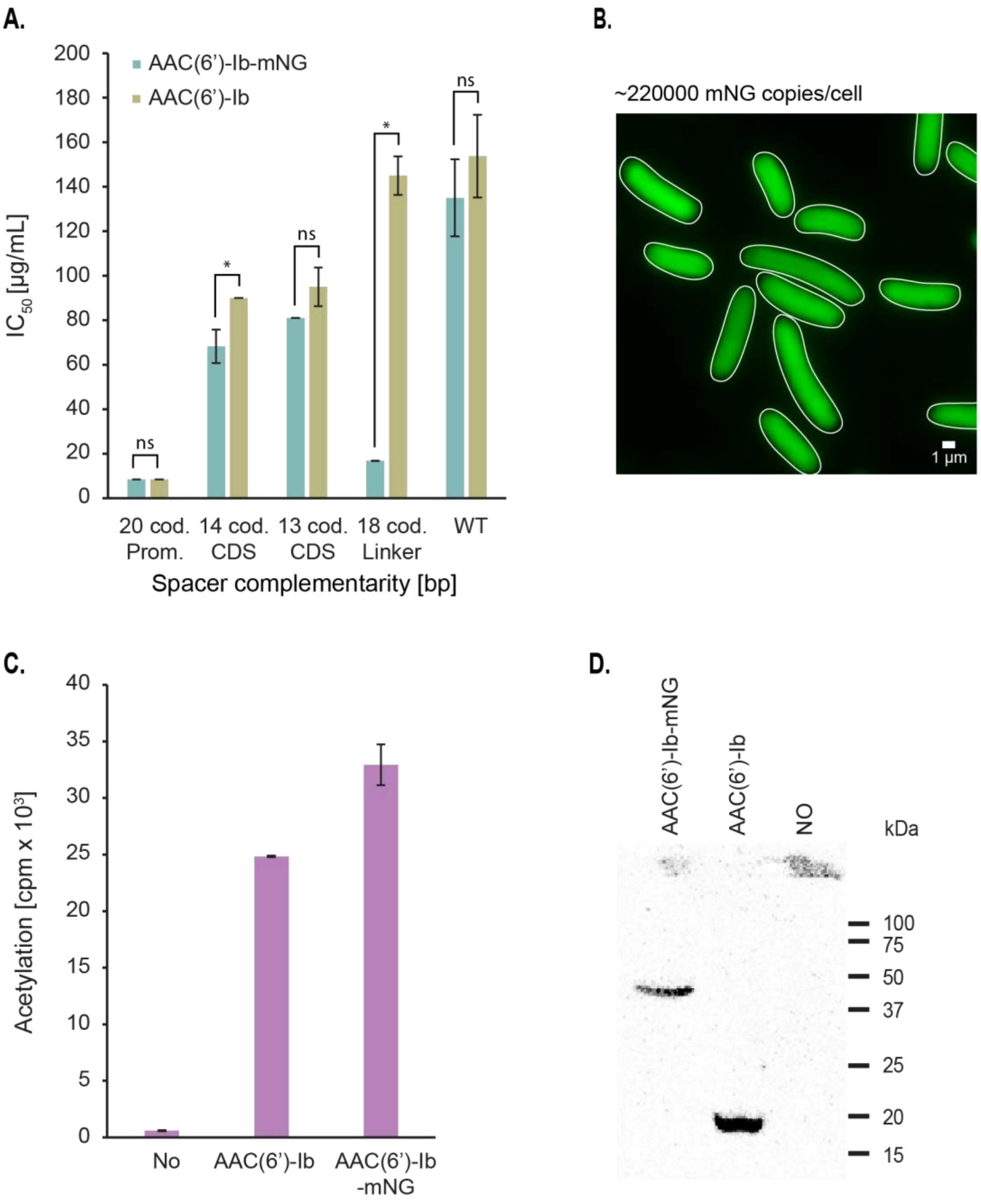

Figure S4 – The mNeonGreen (mNG) fluorescent tag does not alter AAC(6')-Ib activity and does not force protein aggregation. **A.** Inhibitory concentration at which 50% of growth is inhibited by the amikacin antibiotic (IC_{50}) for different repression levels of AAC(6')-Ib copy number per cell. The length of the gRNA that is complementary to the coding (cod.) strand, as well as the targeted region of the *aac(6')-Ib* gene, are indicated (Prom, promoter; CDS, coding sequence; WT, wild-type). Error bars represent the standard deviation and comparative statistical analysis was performed to compare the effect of the mNG tag on the activity of the enzyme in the cell (ns, not significant, and *, significant for

p=0.05). **B.** Representative image of AB1157 cells expressing high concentrations of mNG molecules from a pUC18 derivative plasmid. Copy number quantified by spectrofluorometry (221483.5 ± 16440.8 mNG copies/cell). **C.** Effect of mNG on AAC(6′)-Ib-catalyzed acetylation of amikacin. **D.** Western blot showing total concentration of enzyme, in the wild-type background.

Table S1 - Strains used for this study.

Strain	Relevant genotype	Source
AB1157	*thr-1*, *araC14*, l*euB6*(Am), DE(*gpt-proA*)62, *lacY1*, *tsx33*, qsr'-0, *glnV44*(AS), *galK2*(Oc), LAM$^-$, Rac-0, *hisG4*(Oc), *rfbC1*, *mgl-51*, *rpoS396*(Am), *rpsL31*(str^R), *kdgK51*, *xylA5*, mtl-1, *argE3*(Oc), *thi-1*	(51)
BL21(DE3)	*fhuA2* [lon] *ompT gal* [*dcm*] Δ*hsdS*	(52)
TB25	AB1157 derivative, P_{lacq}-*lacI* $P_{Llac\text{-}s}$-*dCas9 cat::ΔattB*	This work
FHcas1nc	TB25 derivative, *gRNA-Prom (20bp cod.)::argE*	This work
FHcas1	TB25 derivative, *gRNA-Prom (20bp non-cod.)::argE*	This work
FHcas2nc	TB25 derivative, *gRNA-CDS (20bp cod.)::argE*	This work
LW3	TB25 derivative, *gRNA-CDS (15bp cod.)::argE*	This work
TY1	TB25 derivative, *gRNA-CDS (14bp cod.)::argE*	This work
LW2	TB25 derivative, *gRNA-CDS (13bp cod.)::argE*	This work
LW1	TB25 derivative, *gRNA-CDS (12bp cod.)::argE*	This work

TY2	TB25 derivative, *gRNA-CDS (11bp cod.)::argE*	This work
TY3	TB25 derivative, *gRNA-CDS (10bp cod.)::argE*	This work
FHcas2	TB25 derivative, *gRNA-CDS (20bp non-cod.)::argE*	This work
FHcas3nc	TB25 derivative, *gRNA-Linker (18bp cod.)::argE*	This work
TY4	TB25 derivative, *gRNA-Linker (14bp cod.)::argE*	This work
TY5	TB25 derivative, *gRNA-Linker (11bp cod.)::argE*	This work
TY6	TB25 derivative, *gRNA-Linker (10bp cod.)::argE*	This work
LW4	TB25 derivative, *gRNA-Linker (9bp cod.)::argE*	This work
FHcas3	TB25 derivative, *gRNA-Linker (15bp non-cod.)::argE*	This work
OD030	P_{Lac}-*mMaple kan::ΔgalK*	This work
YHZ23	*MATa his3Δ1 leu2Δ0 met15Δ0 LYS2 ura3Δ0 nup59-mNeonGreen-Nat*	This work

Table S2 – Plasmids used in this study.

Plasmid	Description	Source
pTT4	pJHCMW1 derivative containing 96 copies of *tetO* inserted into the *tnpA* gene.	(17)
pTT4-mNG-wL	pTT4 derivative carrying an *aac(6')-Ib-mNeonGreen* fusion.	This work
pFH3	pTT4 derivative carrying an *aac(6')-Ib-mMaple* fusion.	This work
pROD93	Plasmid containing the *mMaple* gene under a P_{Llac} promoter, with an R6K gamma origin and kanamycin resistance.	(36)
pTB35	Plasmid carrying the *dCas9* gene under P_{Llac} promoter with constitutive lacI. Used for *attP* integration with chloramphenicol resistance.	(53)
pTB40-1	Plasmid expressing dnaX-targetting gRNA. R6K gamma *ori*.	This work
pVV03	pUC18 derivative expressing mNeonGreen from a lac promoter and Kanamycin resistance	This work

Table S3 – Primers used in this work. The guide RNA sequences are highlighted in green, while the mutated base pairs are shown in blue. cod., coding, non-cod., non-coding, compl., complementary, CDS, coding sequence.

Primer	Sequence 5'-3'	Description
TB04	ACTAGTAUTATACCTAGGACTGAG	Fixed primer for gRNA mutagenesis on the pTB40-1 plasmid
FHcas1	ATACTAGUTGCTTCAATAATATTGAAAAGTTTTAGAGCTAGAAATAGCAAG	Primer for gRNA mutagenesis of the pTB40-1 plasmid. 20bp compl. to non-cod. strand of promoter.
FHcas1nc	ATACTAGUCAGGGTTATTGTCTCATGAGGTTTTAGAGCTAGAAATAGCAAG	Primer for gRNA mutagenesis of the pTB40-1 plasmid. 20bp compl. to cod. strand of promoter.
FHcas2	ATACTAGUCAACATTTCCAAACAAAGTTGTTTTAGAGCTAGAAATAGCAAG	Primer for gRNA mutagenesis of the pTB40-1 plasmid. 20bp compl. to non-cod. strand of CDS.
FHcas2nc	ATACTAGUTTGTGATGCCTAACTTTGTTGTTTTAGAGCTAGAAATAGCAAG	Primer for gRNA mutagenesis of the pTB40-1 plasmid. 20bp compl. to cod. strand of CDS.
FHcas3	ATACTAGUGCCATGGCTGGCTCCGCTGCGTTTTAGAGCTAGAAATAGCAAG	Primer for gRNA mutagenesis of the pTB40-1 plasmid. 15bp compl. to non-cod. strand of linker.
FHcas3nc	ATACTAGUGACTGCGCCAGAACCAGCAGGTTTTAGAGCTAGAAATAGCAAG	Primer for gRNA mutagenesis of the pTB40-1 plasmid. 18bp compl. to cod. strand of linker.
14bpFHcas2nc	ATACTAGUAACACTTGCCTAACTTTGTTGTTTTAGAGCTAGAAATAGCAAG	Primer for gRNA mutagenesis of the pTB40-1 plasmid. 14bp compl. to cod. strand of CDS.

11bpFHcas2nc	ATACTAGUAACACTACGCTAACTTTGTTGTTTTAGAGCTAGAAATAGCAAG	Primer for gRNA mutagenesis of the pTB40-1 plasmid. 11bp compl. to cod. strand of CDS.
10bpFHcas2nc	ATACTAGUAACACTACGGTAACTTTGTTGTTTTAGAGCTAGAAATAGCAAG	Primer for gRNA mutagenesis of the pTB40-1 plasmid. 10bp compl. to cod. strand of CDS.
14bpFHcas3nc	ATACTAGUCTGACGGCCAGAACCAGCAGGTTTTAGAGCTAGAAATAGCAAG	Primer for gRNA mutagenesis of the pTB40-1 plasmid. 14bp compl. to cod. strand of linker.
11bpFHcas3nc	ATACTAGUCTGACGCGGAGAACCAGCAGGTTTTAGAGCTAGAAATAGCAAG	Primer for gRNA mutagenesis of the pTB40-1 plasmid. 11bp compl. to cod. strand of linker.
10bpFHcas3nc	ATACTAGUCTGACGCGGTGAACCAGCAGGTTTTAGAGCTAGAAATAGCAAG	Primer for gRNA mutagenesis of the pTB40-1 plasmid. 10bp compl. to cod. strand of linker.
12bpFHcas2nc	ATACTAGUAACACTACCCTAACTTTGTTGTTTTAGAGCTAGAAATAGCAAG	Primer for gRNA mutagenesis of the pTB40-1 plasmid. 12bp compl. to cod. strand of CDS.
13bpFHcas2nc	ATACTAGUAACACTAGCCTAACTTTGTTGTTTTAGAGCTAGAAATAGCAAG	Primer for gRNA mutagenesis of the pTB40-1 plasmid. 13bp compl. to cod. strand of CDS.
15bpFHcas2nc	ATACTAGUAACACATGCCTAACTTTGTTGTTTTAGAGCTAGAAATAGCAAG	Primer for gRNA mutagenesis of the pTB40-1 plasmid. 15bp compl. to cod. strand of CDS.
9bpFHcas3nc	ATACTAGUCTGACGCGGTCAACCAGCAGGTTTTAGAGCTAGAAATAGCAAG	Primer for gRNA mutagenesis of the pTB40-1 plasmid. 9bp compl. to cod. strand of linker.
TB200	ATAAATACTGCATGAATATTGATACTATCATGACCAGAGGTGTGTCAACATTTCGCTAAGGATGATTTCTGG	Primer to insert each gRNA into *argE* gene by lambda red.

TB201	CGGATGCGGCGCGAGCGCCTTATCCGGCCTACGTTTTAATGCCAGCATATCCTCCTTAGTTCCTATTCC	Primer to insert each gRNA into *argE* gene by lambda red.
mMapleNcoI_F	TTTCCATGGCTGGCTCCGCTGCTGGTTC	Primer to clone *mMaple* into pTT4 by *Nco*I digestion.
mMapleNcoI_R	TTTCCATGGTTACTTGTACAGCTCGTCCATGC	Primer to clone *mMaple* into pTT4 by *Nco*I digestion.
galK_insF	GTTTGCGCGCAGTCAGCGATATCCATTTTCGCGAATCCGGAGTGTAAGAACGCCCAATACGCAAACCG	Primer to insert *pLac-mMaple* into *galK* gene by lambda red.
galK_insR	CGGCTGACCATCGGGTGCCAGTGCGGGAGTTTCGTTCAGCACTGTCCTGCCTTATGAATATCCTCCTTAG	Primer to insert *pLac-mMaple* into *galK* gene by lambda red.

Table S4 – Number of repeats and total cell count for Figure 3.D

Strain	Treatment	Number of repeats	Total number of cells	Average copy number ± Standard deviation
TY4	/	3	2402.99 ± 852.69	1212
	EDTA	3	1681.9 ± 689.61	1492
TY1	/	3	5193.5 ± 1673.95	1673
	EDTA	3	4299.94 ± 1375.28	1365
FHcas2	/	3	21798.56 ± 6187.83	1633
	EDTA	3	16461.43 ± 4888.06	639
TB25	/	3	68815.57 ± 15122.08	1900
	EDTA	3	67141.91 ± 21968.79	873

References

51. Dewitt SK, Adelberg EA. 1962. The occurrence of a genetic transposition in a strain of *Escherichia coli*. Genetics 47:577-85.

52. Wood WB. 1966. Host specificity of DNA produced by *Escherichia coli*: bacterial mutations affecting the restriction and modification of DNA. J Mol Biol 16:118-33.

53. Soubry N, Wang A, Reyes-Lamothe R. 2019. Replisome activity slowdown after exposure to ultraviolet light in Escherichia coli. Proc Natl Acad Sci U S A 116:11747-11753.
