# Supplementary material for: Dynamics and quantitative contribution of the aminoglycoside 6′-N-acetyltransferase type Ib to amikacin resistance
Source: mSphere. 2024 Feb 14;9(3):e00789-23. doi: 10.1128/msphere.00789-23 (PMC10964403; doi:10.1128/msphere.00789-23)
Supplement: Supplemental Data — Raw data used to produce figures. [file msphere.00789-23-s0002.pdf]

AAC(6')-Ib copy number per cell in strains carrying varying gRNAs.

Strain	Plasmid	AAC(6')-Ib copy number per cell								Average	Standard Deviation
FHcas1nc	pTT4_mNG_wL	85.6	510.1	260.2						285.3	213.4
FHcas1	pTT4_mNG_wL	266.4	917.4	463.4						549.0	333.8
FHcas2nc	pTT4_mNG_wL	600.3	347.6	721.0						556.3	190.5
FHcas3nc	pTT4_mNG_wL	532.0	1356.6	761.6						883.4	425.6
LW3	pTT4_mNG_wL	1058.7	2438.3	2418.8	2328.2	4042.9	2701.4	3979.2		2709.6	1034.3
TY4	pTT4_mNG_wL	2627.7	4464.7	2745.5						3279.3	1028.3
TY5	pTT4_mNG_wL	4726.1	6575.5	6034.8						5778.8	950.9
TY1	pTT4_mNG_wL	6414.4	11607.0	7839.4	10749.6					9152.6	2435.6
LW2	pTT4_mNG_wL	9753.0	9590.9	9782.8						9708.9	103.3
TY6	pTT4_mNG_wL	9170.1	10112.7	10797.5	12808.4	14782.4	11921.9			11598.8	2022.5
LW4	pTT4_mNG_wL	22443.9	32123.3	21664.1	25640.4					25467.9	4758.8
LW1	pTT4_mNG_wL	21332.1	20776.4	20391.6	19664.5	31575.2	24050.3	30235.9	39019.9	25880.7	6999.0
FHcas2	pTT4_mNG_wL	23505.2	35192.9	25131.7	21269.3					26274.8	6152.6
TY2	pTT4_mNG_wL	41218.0	52964.3	40422.6						44868.3	7022.7
FHcas3	pTT4_mNG_wL	64518.7	75083.0	69774.1						69791.9	5282.2
TY3	pTT4_mNG_wL	66690.1	78150.1	97768.8	84899.7	73563.1				80214.3	11848.2
TB25	pTT4_mNG_wL	87008.1	86125.8	92637.6	88864.8	107540.9	90451.9	99197.1	83671.4	91937.2	7880.4
AB1157	pVV03	203607	224887	235955						221483	16440

AAC(6')-Ib copy number per cell in strains carrying varying gRNAs, in presence of 0.5mM EDTA.

Strain	Plasmid	AAC(6')-Ib copy number per cell				Average	Standard Deviation
LW2	pTT4_mNG_wL	43032.9	15810.5	13137.1		23993.5	16542.7
LW1	pTT4_mNG_wL	30235.9	27518.3	22788.1	31204.1	27936.6	3770.3
TY2	pTT4_mNG_wL	24221.5	63950.9	49669.0		45947.2	20124.5
TB25	pTT4_mNG_wL	99197.1	83299.3	100089.7	72180.0	88691.5	13441.2

AAC(6')-Ib copy number per cell in strains carrying varying gRNAs, in presence or absence of 0.5mM EDTA, estimated with the confocal microscope.

Strain	Plasmid	Condition	AAC(6')-Ib copy number per cell				Average	Standard Deviation
TY4	pTT4_mNG_wL	/	2360.0	2316.0	2499.0		2391.7	95.5
		0.5 mM EDTA	1445.0	1737.0	1807.1		1663.0	192.1
TY1	pTT4_mNG_wL	/	5989.0	6484.0	4571.0	4699.3	5435.8	947.8
		0.5 mM EDTA	4367.0	4753.0	3639.0		4253.0	565.7
FHcas2	pTT4_mNG_wL	/	24284.0	20703.0	19493.0		21493.3	2491.4
		0.5 mM EDTA	16988.0	18924.0	4556.7		13489.6	7796.4
TB25	pTT4_mNG_wL	/	70627.0	67488.0	68396.0	68519.7	68757.7	1328.4
		0.5 mM EDTA	52789.0	81503.0	59702.0		64664.7	14986.5

IC50 for strains expressing different gRNAs

Strain	Plasmid	IC_{50}					Average	Standard Deviation
TB25 C-	/	2.5	2.5	2.5			2.5	0.0
FHCas1	pTT4_mNG_wL	7.8	7.8	8.4	5.6	8.4	7.6	1.2
FHCas2nc	pTT4_mNG_wL	8.4	8.4	8.4			8.4	0.0
FHCas1nc	pTT4_mNG_wL	15.6	7.8	5.6	5.6		8.7	4.8
FHCas3nc	pTT4_mNG_wL	16.9	16.9	16.9			16.9	0.0
TY4	pTT4_mNG_wL	15.6	22.5	22.5	33.8		23.6	7.5
TY5	pTT4_mNG_wL	31.3	33.8	22.5	45.0		33.1	9.3
LW3	pTT4_mNG_wL	62.5	81.0	63.0	33.8	45.0	57.1	18.2
TY1	pTT4_mNG_wL	62.5	81.0	63.0	67.5	67.5	68.3	7.5
TY6	pTT4_mNG_wL	81.0	72.0	63.0	63.0		69.8	8.6
LW2	pTT4_mNG_wL	81.0	81.0	81.0			81.0	0.0
LW1	pTT4_mNG_wL	105.0	105.0	105.0			105.0	0.0
FHCas2	pTT4_mNG_wL	105.0	90.0	105.0	120.0		105.0	12.2
TY2	pTT4_mNG_wL	105.0	105.0	90.0	120.0		105.0	12.2
LW4	pTT4_mNG_wL	105.0	105.0	105.0			105.0	0.0
FHCas3	pTT4_mNG_wL	105.0	105.0	105.0			105.0	0.0
TY3	pTT4_mNG_wL	105.0	135.0	120.0	135.0		123.8	14.4
TB25	pTT4_mNG_wL	150.0	120.0	120.0	150.0		135.0	17.3
TB25	pTT4	150.0	150.0	135.0	135.0	135.0	141.0	8.2
FHcas1nc	pTT4	8.4	8.4	8.4	8.4	8.4	7.0	3.4
FHcas3nc	pTT4	150.0	135.0	150.0	145.0		117.7	61.3
TY1	pTT4	90.0	90.0	90.0	90.0		72.0	40.2
LW2	pTT4	105.0	90.0	90.0	95.0		77.7	39.1
TB25	pTT4	175.0	175.0	175.0	175.0		140.0	78.3

IC_{50} for strains expressing different gRNAs grown with 0.5mM EDTA

**Strain**	**Plasmid**	**IC_{50}**								**Average**	**Standard Deviation**
TB25	/	5.0	5.0	5.0						5.0	0.0
FHcas2nc	pTT4_mNG_wL	5.6	8.4	4.2	5.6	5.6				5.9	1.5
FHcas1	pTT4_mNG_wL	8.4	11.3	5.6	4.2	8.4				7.6	2.7
FHcas1nc	pTT4_mNG_wL	11.3	11.3	8.4	8.4	8.4				9.6	1.5
FHcas3nc	pTT4_mNG_wL	16.9	22.5	11.3	11.3					15.5	5.4
TY5	pTT4_mNG_wL	22.5	22.5	22.5						22.5	0.0
TY4	pTT4_mNG_wL	22.5	22.5	22.5						22.5	0.0
LW3	pTT4_mNG_wL	45.0	33.8	22.5						33.8	11.3
TY6	pTT4_mNG_wL	33.8	33.8	33.8						33.8	0.0
TY1	pTT4_mNG_wL	45.0	45.0	45.0						45.0	0.0
LW2	pTT4_mNG_wL	81.0	81.0	120.0						94.0	22.5
LW4	pTT4_mNG_wL	90.0	120.0	150.0						120.0	30.0
FHcas2	pTT4_mNG_wL	105.0	120.0	175.0						133.3	36.9
LW1	pTT4_mNG_wL	120.0	120.0	120.0	175.0					133.8	27.5
TY2	pTT4_mNG_wL	90.0	150.0	175.0						138.3	43.7
FHcas3	pTT4_mNG_wL	135.0	150.0	175.0						153.3	20.2
TY3	pTT4_mNG_wL	150.0	150.0	200.0						166.7	28.9
TB25	pTT4_mNG_wL	200.0	200.0	200.0						200.0	0.0
FHcas1nc	pTT4	8.4	8.4	8.4	8.4					8.4	0.0
TY1	pTT4	90.0	90.0	90.0						90.0	0.0
LW2	pTT4	105.0	90.0	90.0						95.0	8.7
FHcas3nc	pTT4	150.0	135.0	150.0						145.0	8.7
TB25	pTT4	175.0	175.0	175.0	150.0	150.0	135.0	135.0	135.0	153.8	18.7
